# Through the Second Wave: Analysis of the Psychological and Perceptive Changes in the Italian Population during the COVID-19 Pandemic

**DOI:** 10.3390/ijerph19031635

**Published:** 2022-01-31

**Authors:** Andrea Guazzini, Andrea Pesce, Lorena Marotta, Mirko Duradoni

**Affiliations:** 1 Department of Education, Literatures, Intercultural Studies, Languages and Psychology, University of Florence, 50135 Florence, Italy; andrea.guazzini@unifi.it (A.G.); andrea.pesce@stud.unifi.it (A.P.); lorena.marotta@stud.unifi.it (L.M.); 2 Centre for the Study of Complex Dynamics, University of Florence, 50019 Sesto Fiorentino, Italy

**Keywords:** COVID-19, self-efficacy, locus of control, risk perception, habituation

## Abstract

More than a year has passed since “patient 0” was found and COVID-19 is now an established reality that a lot of people have had to accept and coexist with. In Italy, the pandemic hit in “waves”, but the studies assessing the longitudinal impact of the pandemic in the general population are not many. In this paper, we compared data collected during the first wave with data gathered during the second one, so that we can provide insights about the Italian population’s psychological adaptation to the pandemic also by comparing them with pre-pandemic normative scores. From our results, it seemed that people were seeking a compromise: indeed, despite the still-relevant risk perception, people apparently “learned” how to deal with the pandemic as indicated by an increase in self-efficacy and a more internal locus of control.

## 1. Introduction

Since December 2019 and until January 2021, the outbreak of SARS-CoV-2 has led to a pandemic of illness (COVID-19) that has caused 226,236,577 million cases and 4,654,548 deaths worldwide [1,2]. The containment measures and quarantine periods implemented by the various governments around the world had an impact on the psychological and psychosocial equilibria of all the communities around the world, and they continue to affect day-to-day life [3,4].

Italy, after China, was one of the first countries to be severely affected by the diffusion of the virus and to implement these measures: the first lockdown started in mid-March 2020 and ended in late May of the same year [5]. In June, the country mostly reopened. The number of cases was initially extremely low, even if the virus never really disappeared, and by the end of summer 2020 contagion numbers were increasing again. This is when the second wave started in Italy, and with it a new series of restrictions and preventive measures were established [6].

On 3 November 2020, the Italian government issued diversified measures, depending on risk indicators (e.g., positive COVID tests, hospital overload) that were defined by the Italian Ministry of Health: in particular, regions of Italy were categorized in three risk clusters, yellow, orange, and red. Respectively, yellow regions have a lower risk of diffusion, orange regions have a medium-high risk, and red regions have a high risk of diffusion [1,7,8].

From the analysis of literature, we can see how restrictions, economic uncertainty, and doubts and fears for the future had a strong impact on mental health and other psychological variables [9,10,11,12]. In Italy, quarantine measures had been modeled after China’s, and therefore were especially strict. This had a strong impact on the mental health of people, especially in regards to well-being, negative emotions, and psychological distress [9,13]. Moreover, effects due to the long exposure to the emergency situation (e.g., suicidal tendencies, anxiety, languishing) were reported during the first wave [14,15].

Comparing Italy to other European countries, we observed that the second wave of the pandemic seemed to have had a lower impact on mental health [16]. Emotional regulation and coping strategies were also protective factors, because they allowed for a re-appraisal of the situation, and they modified the intensity and the duration of negative emotions [17,18]. Literature suggested that most people were resilient, but that it is also important to understand which factors came into play to promote habituation [19].

More than a year has passed since “patient 0” was found, and while the pandemic does not seem to be stopping yet, people have lowered their guard and are trying to go back to the “normal” way of life despite all the COVID-related risks that are still very present. It looks like the prolonged exposure to the danger might have lowered the risk perception. The desire for freedom with the subsequent lower compliance to the same norms that were so successful during the first lockdown can be explained in terms of psychological reactance [20]. It is also worth wondering how mental health and psychological variables were impacted by the second wave after that brief moment of normalcy between the two lockdowns, i.e., if they got worse, better, or if they remained stable. We have noticed, however, an almost complete lack of studies investigating the comparison between the first and the second wave, especially regarding Italy.

This study wanted to compare data collected during the first wave with data gathered during the second one to establish a trajectory of specific psychological dimensions during the pandemic, so that we can provide a general framework of mental health in the Italian population. While other studies generally examined discomfort and negative emotions [9,12,13], our study wanted to provide a more comprehensive assessment of psychological and perceptive changes (i.e., thus not including just “negatively” framed psychological variables such as anxiety and depression) in the transition from the first to the second wave. These can become a key factor to understand the underlying mechanisms to habituation and how people have adapted their behaviors to coexist with COVID-19.

## 2. Method

### 2.1. Sample and Procedure

To assess the changes in the psychological dimensions presented by Marotta et al. [21] during the second wave in Italy (*N* = 1556), we conducted a cross-sectional survey among the general public in Italy (*N* = 501) through an anonymous online questionnaire. The participants in the study were selected through a snowball sampling as in the previous study [22]. Participants gave their informed consent and completed the survey via the Google form online platform. The questionnaires were administered to the participants accordingly to the Italian law’s requirements of privacy and informed consent (Law Decree DL-101/2018), EU regulation (2016/699), and (APA) guidelines. Moreover, expedited approval was obtained from the University of Florence’s ethical commission (approved protocol number: 0092811). In the end, two samples were involved in this study: the first came from the work of Marotta et al. [21] (data were collected from 6 April to 12 April 2020) while the second sample was recruited between 23 October 2020 and 20 November 2020. Part of the data of this second sample were used in Guazzini et al. [23].

### 2.2. Materials

The authors aimed to assess the psychological impact of the second COVID-19 wave by comparing the data gathered by Marotta et al. [21] during the first wave with those related to the second. In order to allow for a fair comparison, the authors decided to rely on the same poll of measures selected by Marotta et al. [21].

The adopted measures were grouped in five categories: “Well-being”, which includes the Flourishing Scale, Warwick-Edinburgh Mental Well-Being Scale (WEMWBS), and Satisfaction with Life; “Social”, which includes Sense of Virtual Community and Social Connectedness; “Self”, which includes Locus of Control (LOC), Self-Efficacy, and General Risk Propensity Scale (GRiPS); “Affects”, which includes Positive Affect and Negative Affect Scale (PANAS); and “Risk Perception”, which includes the Wilson Questionnaire.

#### 2.2.1. Well-Being

To assess well-being, we used the following scales:

We used the Flourishing Scale (FS) by Diener et al. [24] to measure eudaimonic well-being. The Flourishing Scale is a brief 8-item summary measurement of the respondent’s self-perceived success in important areas such as relationships, self-esteem, purpose, and optimism. The scale provides a single psychological well-being score. Replies go from 1 to 7 for all eight items, and the scores are summed together. The possible range of scores is from 8 to 56. The internal reliability coefficient is α= 0.90.

We relied on the Satisfaction with Life Scale (SWLS) by Diener et al. [25] to measure hedonic/affective well-being. It is a 5-item scale designed to measure global cognitive judgments of one’s life satisfaction. Participants indicate how much they agree or disagree with each of the 5 items on a 7-point scale: the first three items refer to present satisfaction, and the other two to the past. The possible range of scores is from 5 to 35. Its internal reliability coefficient is α = 0.85

We used the Warwick-Edinburgh Mental Well-Being Scale (WEMWBS) [26]; the WEMWBS is a 12-item scale of mental well-being covering subjective well-being and psychological functioning, in which all items are worded positively, and they all address aspects of positive mental health. The scale is scored by summing responses to each item answered on a 1 to 5 Likert scale. The 12-item scale has 5 response categories, summed to provide a single score. Scores can range from 12 to 60 points. Its internal reliability coefficient is α = 0.91.

#### 2.2.2. Social

To assess social related dimensions, we used the following:

Social connectedness was measured through the Social Connectedness Scale-Revised (SCS-R) by Lee and Robbins [27]. The SCS-R 16 comprises 20 items on a 6-point scale assessing experiences of closeness in interpersonal contexts, as well as difficulties establishing and maintaining a sense of closeness. Ten items are negatively worded while the remaining are positively worded. Negative items are reverse scored, and a higher score indicates a greater social connectedness: the scores can range from 20 to 120. Its internal reliability coefficient is α = 0.55.

We used the Sense of Virtual Community Scale (SoVc) by Blanchard [28] to measure the sense of community in virtual environments. It is an 18-item scale on a four-point Likert scale, and it measures one’s sense of belonging in a virtual community. The possible range of scores is from 18 to 72. Its internal reliability is α = 0.60.

#### 2.2.3. Self

To assess self-related dimensions, we used the following:

We used the Locus of Control of Behavior (LCB) by Craig et al. [29] to measure the locus of control. The locus of control refers to the extent to which people feel that they have control over the events that influence their lives. A lower score in this questionnaire means that the locus of control is internal. It consists of 17 items measured on a 6-point scale and the score is calculated by adding the items that are related to the external locus of control and subtracting the ones that are related to an internal locus of control. The LCB possible range of scores is 0–85. Its internal reliability is α = 0.71.

Self-efficacy was measured using the Generalized Self Efficacy Scale (GSE) by Schwarzer and Jerusalem [30]. The Generalized Self-Efficacy Scale is a 10-item psychometric scale on a 5-point Likert scale that is designed to assess optimistic self-beliefs to cope with a variety of difficult demands in life. The score is calculated by adding together the answers to all the items: the higher the score, the higher the self-efficacy. Scores can range between 10 and 50. Its internal reliability coefficient is α = 0.75.

Risk propensity was measured using the General Risk Propensity Scale (GRiPS) by Zhang et al. [31]. This is a unidimensional, self-report measurement of general risk propensity. This scale is 8 items and measures people’s general propensity to take risks across situations on a 5-point scale. The possible range of scores is from 1 to 5. Its internal reliability is α = 0.90.

#### 2.2.4. Affects

To assess affect-related dimensions, we relied on the following measures:

To assess dimensions related to affects, we used the Positive and Negative Affect Schedule (PANAS) by Watson et al. [32]. The PANAS is a scale that consists of different words that describe feelings and emotions. This scale consists of 20 items, with 10 items measuring positive affect (e.g., excited, inspired) and 10 items measuring negative affect (e.g., upset, afraid). Each item is rated on a five-point Likert scale. Scores can range from 10 to 50 for both positive and negative. Its internal reliability is α = 0.76.

We measured risk perception using the scale created by Wilson et al. [33]. Perceived risk was elicited by nine items graded on a 5-point Likert-type scale (0 = not at all, 5 = extremely). The scale measures risk perception as a multidimensional construct composed by general (range 1–5), probability (range 2–10), consequences (range 2–10), and affect (range 5–25) dimensions. In the study by Ding and colleagues [34], the internal reliability is α = 0.64.

### 2.3. Data Analysis

Before the recruitment of the second sample to be compared with the first one coming from the work by Marotta et al. [21], we performed a power analysis to establish an adequate sample size for our research purposes. We used G*Power software to accomplish this procedure [35,36]. This analysis was performed taking into account that the number of the first sample was already fixed at 1556. For testing wave-related differences (*t*-test), a sample of 1054 individuals would be required to reach a statistical power of 0.80, supposing a small effect size (d = 0.20) and an expected wave ratio of 1:0.33 (i.e., group 1 = 792; group 2 = 262). Since the composite final sample consisted of 2057 individuals, the authors considered the sample size suitable for carrying out the analyses.

## 3. Results

As a first step, we provided a description of the sample according to the selected sociodemographic and psychological variables (Table 1), and we checked for the preconditions necessary for the parametric inferential analysis through the assessment of asymmetry and kurtosis values for normality, by scatterplots of residuals versus predicted values for homoscedasticity, and by means of simple scatterplots for linearity.

Subsequently, we analyzed the relationships between target psychological variables and both discrete and continuous sociodemographic factors. We did this to understand whether sociodemographic variables should be considered or excluded in exploring effects due to the time elapsed between the two waves since we were not comparing a proper paired sample but two distinct samples. A power analysis was run for each type of statistical analysis the authors planned to perform. For ANOVA, since the authors were interested in detecting effect sizes higher than 5%, they assumed prudentially an f = 0.20 (that is roughly equal to 4% effect size). The power analyses showed that a sample size of 200 individuals (for the sex variable) and 330 (for the occupation variable) would be enough to ensure a statistical power of 0.80 (significance level 0.05), to assess sex-related and occupation-related differences. At the same time, Pearson’s r correlation was planned for assessing the correlation between the collected data and the continuous sociodemographic variables. For Pearson’s correlation (bivariate normal model), a sample of 193 would be required to achieve the same statistical power (i.e., 0.80), while assuming the same effect size (r = 0.20; 4% effect size). Thus, our sample size was deemed as adequate.

As highlighted by the ANOVA tests, both sex and occupation (Table A1) did not appear to considerably affect our criterion variables. In the case of statistically significant results, the effect size (η^2^) was always below 5%. Similar results were obtained exploring the effects of continuous variables (i.e., age and education) through Pearson’s correlation (Table A2). Even in this case, when existent, the effect never exceeded 5%. For this reason, we decided to exclude sociodemographic factors as possible confounding variables to be controlled for when analyzing differences between waves on our criterion variables.

We relied on Welch’s *t*-test to assess whether our criterion variables showed a significant difference between the first and second wave and, in the case of statistically significant results, we computed Cohen’s d as the effect size measurement. Cohen’s d values of 0.2, 0.5, and 0.8 have been identified by Cohen [37] as, respectively, small, medium, and large effect sizes.

As we can gather from Table 2, the locus of control appeared as one of the most affected variables in our pool. Participants in the second wave reported much higher levels of the internal locus of control than people recruited during the first wave. In addition, the perceived likelihood of infection (Wilson probability) and general risk perception appeared as considerably affected. The second wave seemed to be characterized by a higher perception of risk in this sense. The other eight variables showed statistically significant differences. More specifically, the second wave appeared to be characterized by slightly higher levels of self-efficacy, risk propensity (GRIPS), sense of virtual community (SoVC), social connectedness, mental well-being (WEMWBS), cognitive hedonic well-being (SWL), and by an increase in positive affectivity (PANAS Positive) and a decrease in negative affectivity (PANAS Negative). A more in-depth analysis of the variations related to the single emotions covered by the PANAS is shown in Table A3. No significant differences were found regarding eudaimonic well-being (flourishing) and two components of risk perception, namely affect and consequences.

**Figure 1:**
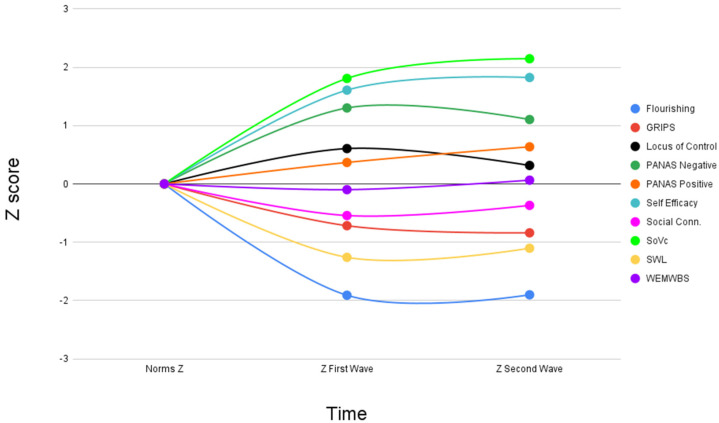
Trajectory of psychological criterion variables from pre-pandemic to November 2020 expressed in Z-points representing how far from the pre-pandemic mean a data point is. In the figure, lower scores in GRIPS highlight higher levels of risk propensity.

Finally, we tried to picture the full trajectory of our criterion variables from pre-pandemic to November 2020 passing through the first wave (Figure 1).

In Figure 1, for all the variables that had pre-pandemic normative values (Wilson risk perception did not have them), we were able to trace the score deviation from the pre-pandemic situation and monitor it along the two waves. In general, except for the locus of control which marked a clear reversal towards normative and pre-pandemic values, the variables most impacted in the second wave were the same as the first wave (SoVC, self-efficacy, flourishing) with deviations of Z-points close to |2|.

## 4. Discussion

The objective of our study was to assess the differences between the first COVID-19 wave in Italy and the second one, so that we could have a better understanding of the psychological variables’ trajectory in this period of time. Our work allowed us to observe how the Italian population reacted to this unprecedented situation. After conducting a global analysis on the effects of the pandemic (i.e., assessing data from both the first and the second wave and comparing them with pre-pandemic normative data), we observed the following:

Well-Being is generally one of the most impacted factors. Particularly, Flourishing (eudaimonic well-being) and Satisfaction with Life (hedonic-cognitive well-being) scores captured during the second wave seemed to still be much lower than pre-pandemic values (Flourishing: 38.33 before the pandemic, 20.71 in the second wave; SWL: 23.28 before the pandemic, 16.71 in the second wave). Results related to negative emotional activation (hedonic-affective well-being) too seemed to support the fact that the pandemic could have caused a lower well-being. Regarding people’s emotional activation, we also observed an improvement in PANAS scores from the first to the second wave despite its small magnitude (PANAS Negative: 24.07 in the first wave, 22.84 in the second). The WEMWBS, instead, did not show a significant variation due to the pandemic, while positive emotional activation linearly increased, although with a quite flat slope, until the beginning of the second wave of the pandemic (PANAS Positive: 30.17 in the first wave, 32.05 in the second). Generally speaking, of the five well-being measures examined, three were strongly impacted in a negative sense [38,39]. The well-being worsening across the two waves in Italy may allow us also to better contextualize other Covid-19 related outcomes and phenomena, like vaccination hesitancy. Indeed, lower well-being appeared connected to belief in conspiracy theories and mistrust in medicine and science, which ultimately may lead to avoiding vaccination [40,41]. 

Another aspect that seemed to be greatly impacted by the pandemic is SoVC. People seemed to have compensated for the absence of real-life social contact with virtual environments. During the first lockdown, the feeling of deprivation of social “physical” contact was compensated by an increased usage of communication technologies [21,42]. However, even if people were slowly going back to an increasingly more social day-to-day life during the second wave (as indicated by the social connectedness increase between the two waves), the SoVC was still higher than it was before the pandemic, and it was even reported as growing from the first wave to the second (SoVC 27.01 before the pandemic, 43.27 in the second wave). As containment measures became less and less strict, people started seeking real life connections along with virtual connections, following their need to go back to a more “traditional” way of experiencing relationships and interaction. The virtual sense of community, however, did not stop being important and plausibly people discovered that ICTs can be a functional way to get around limitations and to cultivate relationships and shared interests even in times where face-to-face interactions are not fully impeded [43].

The impacts of some specific psychological variables were then assessed by comparing their data between the first and second wave: doing so, it was possible to estimate how people reacted and adapted to this unprecedented situation. In particular, risk propension seemed to have increased in the comparison between the first and the second wave (GRISP: 26.93 in the first wave, 27.84 in the second). These results might suggest a habituation dynamic, in which a perpetual exposure to a risk tends to decrease the anxiety of people towards the risk [44,45], thus making people more inclined to take risks [46,47,48]. This can happen even when risk perception appears to have increased (Wilson probability: 6.68 in the first wave, 8.06 in the second), as habituation can be mediated by other factors like increased self-efficacy or locus of control [49,50]. In fact, in our findings self-efficacy also showed an interesting trend, with a progressive increase from before to during the pandemic (Self-Efficacy: 28.28 before the pandemic, 36.54 in the second wave). A higher sense of self-efficacy seems to also be accompanied by a more internal locus of control: as explained by Rosenstock and colleagues [51], people with high self-efficacy tend to follow professional health advice more often if they also have a more internal locus of control. Locus of control, indeed, showed a small-medium modification in our data (Locus of Control: 27 before the pandemic, 32.57 in the first wave, 29.91 in the second wave), and this might suggest that people have developed a stronger perception of control on their own reality (so, a more internal locus of control) as the pandemic continued to persist. Schurer [52] suggested that when people have a more internal locus of control, they also tend to experience fewer negative emotions (as in our case in which negative affection levels decreased between waves); therefore, they also better endure adverse situations [52,53,54]. We think that the fact that the locus of control has become more internal as the COVID-19 pandemic continued is quite an interesting result: to our knowledge, this cannot be said for other pandemics outside of COVID-19. News about vaccination availability could have affected this result; however, our data were collected between 23 October 2020 and 20 November 2020, thus approximately one month earlier than the official start of the vaccination campaign in Italy (i.e., 27 December 2020). For this reason, we speculate that the internalization of the locus of control might be more related to the fact that, at the time of the study, people had to deal with containment and prevention measures for almost a year: this means that they had more than enough time to “practice” useful habits to avoid the spread of the contagion, thus making the locus of control more internal. The result about the locus of control internalization across the two waves may shed a light, once again, on the outcome of the vaccination campaign in Italy that began shortly thereafter. A more external locus of control was reported as directly associated with hesitant or negative vaccination intentions [55].

Presently, the pandemic is an established reality that a lot of people have had to accept and coexist with. This could explain why, despite the risk perception still being relatively high in our data, we also found a slight increase in risk propensity: people are seeking for a compromise to go back to the pre-pandemic way of life; therefore, they learn and internalize which behaviors they should enact to deal with the virus and they feel generally more self-efficient and they believe in their own abilities to face the situation [56], in accordance with the Self-Perception Theory as postulated by Bem [57]. This is also supported by the Extended Parallel Process Model [58]: together, a higher self-efficacy and a higher perceived threat can lead to danger control processes. A limitation of our study is that the increase in the familywise error rate across the reported statistical analyses was not controlled. Overall, we consider our results to be preliminary and encourage replication.

## 5. Conclusions

A situation becomes “quotidianised” when people begin to take it for granted, as an implied and natural part of everyday life [59]. The COVID-19 emergency is an unprecedented situation that for more than a year has brought huge changes to everyone’s life. An initial and drastic change in routine that had a strong impact on people’s well-being, emotions, and capability of establishing new social connections through virtual environments [21] was followed by a slow process of getting used to the changes [60]. In Italy, people adapted to a condition that was no longer a transitory emergency as in the beginning but a fairly stable reality of coexistence and exposure to the virus, by increasing their level of self-efficacy and internal locus of control.

## Figures and Tables

**Table 1:** Descriptive statistics of the collected data.

Continuous Variables	Pre-Pandemic Norms (Standard Deviation)	Wave I (*N* = 1556)		Wave II (*N* = 501)	
		Min-Max	Mean (Standard Deviation)	Min-Max	Mean (Standard Deviation)
Age	-	15–83	27.88 (10.33)	18–74	34.61 (14.14)
Education (years)	-	8–21	14.48 (2.80)	5–21	15.43 (2.94)
Flourishing	38.33 (9.27)	8–40	20.46 (8.08)	8–40	20.71 (8.34)
Satisfaction with Life Scale	23.28 (5.96)	5–25	15.78 (4.62)	5–25	16.71 (4.54)
WEMWBS	42.06 (6.59)	12–60	41.41 (6.63)	21–56	42.48 (5.88)
Social Connectedness	91.00 (13.83)	22–120	83.50 (16.69)	29–119	85.91 (16.15)
Sense of Virtual Community	27.01 (7.58)	18–72	40.71 (14.81)	18–72	43.27 (13.63)
Locus of Control	27.00 (9.20)	5–58	32.57 (9.19)	6–54	29.91 (8.77)
Self-Efficacy	28.28 (4.53)	10–50	35.56 (6.50)	11–50	36.54 (6.05)
GRiPS	2.3 (0.93)	1–5	1.63 (1.12)	1–5	1.52 (1.19)
PANAS (Positive)	27.60 (7.00)	10–50	30.17 (7.36)	12–50	32.05 (6.35)
PANAS (Negative)	16.00 (6.20)	10–49	24.07 (7.15)	10–46	22.84 (6.33)
Wilson General	Not available	1–5	2.61 (0.95)	1–5	3.17 (0.93)
Wilson Affect	Not available	5–25	16.18 (4.74)	5–25	15.83 (4.50)
Wilson Probability	Not available	2–10	6.68 (1.78)	2–10	8.06 (1.54)
Wilson Consequences	Not available	2–10	6.23 (2.15)	2-10	6.41 (2.05)
**Discrete Variables**		**%**		**%**	
Sex					
Male		16.3%		21.7%	
Female		83.7%		78.3%	
Occupation					
Unemployed		15.1%		9.6%	
Student		48.4%		37.9%	
Autonomous worker		10.0%		25.0%	
Permanent worker		19.4%		19.4%	
Public employee		5.4%		4.6%	
Retired		1.7%		3.6%	

**Table 2:** Differences between first and second waves in Italy.

Variables	*t* *	*p*-Value	df	Cohen *d*
Self Efficacy	−3.11	0.002	900.52	−0.16
Locus of Control	5.84	<0.001	880.40	0.29
GRIPS	−1.87	0.05	804.63	−0.10
SoVc	−3.58	<0.001	910.59	−0.18
Social Connectedness	−2.87	0.004	869.53	−0.15
Flourishing	−0.17	0.88	823.79	NC
WEMWBS	−3.44	0.001	942.22	−0.17
SWL	−3.97	<0.001	858.65	−0.20
PANAS Positive	−5.55	<0.001	968.82	−0.27
PANAS Negative	3.64	<0.001	943.84	0.18
Wilson General	−11.80	<0.001	862.32	−0.59
Wilson Affect	1.48	0.14	883.88	NC
Wilson Probability	−16.78	<0.001	963.26	−0.83
Wilson Consequences	−1.66	0.10	852.56	NC

**Note:***t* *: Welch’s *t*-test; N_(1)_: 1556, N_(2)_: 501; NC = not computed due to a non-statistically significant result; GRIPS = General Risk Propensity Scale; SoVc = Sense of Virtual Community; WEMWBS = Warwick-Edinburgh Mental Well-Being Scale; SWL = Satisfaction with Life.

## Data Availability

The data presented in this study are available on request from the corresponding author.

## Appendix A

**Table A1:** Sex and occupation related differences in collected variables.

Variable	Sex		Occupation	
	Wave I	Wave II	Wave I	Wave II
	F(η^2^)	F(η^2^)	F(η^2^)	F(η^2^)
Self-Efficacy	38.84 *** (2.4%)	NS	5.48 *** (1%)	3.45 * (2%)
Locus of Control	NS	NS	NS	NS
GRIPS	10.43 *** (0.7%)	6.7 ** (1.3%)	3.60 * (0.7%)	NS
SoVC	6.71 ** (0.4%)	NS	NS	3.98 ** (2.3%)
Social Conn.	NS	14.82 *** (2.9%)	NS	NS
Flourishing	21.00 *** (1.3%)	NS	NS	NS
WEMWBS	14.20 *** (0.9%)	NS	6.56 *** (1.3%)	4.17 ** (2.5%)
SWL	NS	NS	2.98 * (0.6%)	NS
PANAS POS	39.74 *** (2.5%)	NS	5.51 *** (1.1%)	NS
PANAS Neg	45.78 *** (2.9%)	6.01 * (1,2%)	15.08 *** (2.8%)	7.68 *** (4.4%)
Wilson General	NS	NS	11.12 *** (2.1%)	NS
Wilson Affect	76.19 *** (4.7%)	17.29 *** (3.4%)	NS	NS
Wilson Probability	22.90 *** (1.5%)	4.79 * (1%)	NS	2.62 * (1.6%)
Wilson Consequences	7.57 ** (0.5%)	NS	2.68 * (0.5%)	NS

**Note:** N_(1)_: 1556, N_(2)_: 501; *: *p* < 0.05; **: *p* < 0.01; ***: *p* < 0.001. NS = not statistically significant result; GRIPS = General Risk Propensity Scale; SoVc = Sense of Virtual Community; WEMWBS = Warwick-Edinburgh Mental Well-Being Scale; SWL = Satisfaction with Life.

**Table A2:** Age and education related correlation in collected variables.

Variable	Age		Education	
	Wave I	Wave II	Wave I	Wave II
	*r*(*r*^2^)	*r*(*r*^2^)	*r*(*r*^2^)	*r*(*r*^2^)
Self-Efficacy	0.12 *** (1.4%)	NS	0.10 *** (1%)	0.13 *** (1.7%)
Locus of Control	NS	0.09 * (0.6%)	−0.08 * (0.6%)	−0.15 *** (2.2%)
GRIPS	0.11 *** (1.2%)	NS	NS	−0.10 * (1%)
SoVC	NS	NS	0.05 * (0.2%)	0.18 *** (3.2%)
Social Conn.	0.05 * (0.2%)	NS	0.12 *** (1.4%)	0.10 * (1%)
Flourishing	−0.08 *** (0.6%)	NS	NS	NS
WEMWBS	0.10 *** (1%)	0.13 *** (1.7%)	0.07 ** (0.5%)	0.12 ** (1.4%)
SWL	NS	NS	0.08 *** (0.6%)	0.11 ** (1.2%)
PANAS POS	NS	−0.10 * (1%)	0.11 *** (1.2%)	0.11 ** (1.2%)
PANAS Neg	−0.17 *** (2.9%)	−0.21 *** (4.4%)	−0.10 *** (1%)	NS
Wilson General	0.08 *** (0.6%)	−0.09 * (0.8%)	NS	0.12 ** (1.4%)
Wilson Affect	0.11 *** (1.2%)	NS	NS	NS
Wilson Probability	NS	−0.16 *** (2.6%)	NS	0.16 *** (2.6%)
Wilson Consequences	0.12 *** (1.4%)	0.16 *** (2.6%)	NS	NS

**Note:** N_(1)_: 1556, N_(2)_: 501; *: *p* < 0.05; **: *p* < 0.01; ***: *p* < 0.001. NS = not statistically significant result; GRIPS = General Risk Propensity Scale; SoVc = Sense of Virtual Community; WEMWBS = Warwick-Edinburgh Mental Well-Being Scale; SWL = Satisfaction with Life.

**Table A3:** Variations across waves in PANAS single emotions.

PANAS Emotion	Wave	M	se	*t* *	df	Cohen *d*
Determined	I	3.21	0.02	−4.16 ***	891	−0.21
	II	3.41	0.04			
Active	I	3.10	0.03	−3.67 ***	871.55	−0.18
	II	3.28	0.04			
Interested	I	3.40	0.04	−4.73 ***	959.18	−0.23
	II	3.61	0.03			
Attentive	I	3.31	0.03	−5.43 ***	921.93	−0.26
	II	3.56	0.04			
Enthusiastic	I	2.81	0.03	−4.30 ***	920.83	−0.22
	II	3.03	0.04			
Concentrating	I	2.90	0.03	−6.62 ***	916.26	−0.33
	II	3.22	0.04			
Strong	I	3.03	0.03	−2.66 **	940.12	−0.13
	II	3.15	0.04			
Inspired	I	2.94	0.03	NS		
	II	2.98	0.04			
Excited	I	2.51	0.03	−3.55 ***	891.22	−0.18
	II	2.70	0.05			
Proud	I	2.96	0.03	−2.55 **	957.66	−0.13
	II	3.10	0.05			
Afraid	I	2.77	0.03	NS		
	II	2.73	0.04			
Upset	I	2.82	0.03	4.14 ***	964.08	0.20
	II	2.62	0.04			
Nervous	I	2.97	0.03	3.69 ***	886.58	0.18
	II	2.78	0.05			
Jittery	I	2.68	0.03	NS		
	II	2.60	0.05			
Scared	I	2.47	0.03	1.96 *	952.09	0.10
	II	2.37	0.04			
Distressed	I	2.60	0.03	4.00 ***	892.78	0.19
	II	2.38	0.05			
Guilty	I	1.50	0.02	−1.92 *	849.72	−0.10
	II	1.58	0.04			
Ashamed	I	1.50	0.02	NS		
	II	1.53	0.04			
Irritable	I	2.87	0.03	4.95 ***	948.23	0.25
	II	2.61	0.04			
Hostile	I	1.88	0.03	NS		
	II	1.64	0.04			

**Note:***t* *: Welch’s *t*-test; N_(1)_: 1556, N_(2)_: 501; se: standard error *: *p* < 0.05; **: *p* < 0.01; ***: *p* < 0.001. NS = not statistically significant result.
